# Knowledge of Health Students Regarding Nutritional Deficiencies in Patients With Celiac Disease in Jazan Region: A Cross-Sectional Study

**DOI:** 10.7759/cureus.62558

**Published:** 2024-06-17

**Authors:** Hussein Ageely, Samar M Alfaifi, Faisal Abusageah, Basem Zogel, Sawsan Alsharif, Mariam Tawhari, Sulaiman Hamdi, Yumna Abutalib, Sultan Althurwi, Lamees Zaalah, Hassan Moafa, Alhassan H Hobani, Ali Mohammed Someili, Ali M Kariri

**Affiliations:** 1 Internal Medicine, Faculty of Medicine, Jazan University, Jazan, SAU; 2 Medicine and Surgery, Jazan University, Jazan, SAU; 3 Tropical Medicine, College of Public Health and Tropical Medicine, Jazan University, Jazan, SAU; 4 Internal Medicine and Gastroenterology, Faculty of Medicine, Jazan, SAU; 5 Internal Medicine, Jazan University, Jazan, SAU

**Keywords:** health student, saudi arabia, celiac disease, medical education, nutritional deficiencies

## Abstract

Introduction: In Saudi Arabia, the prevalence of celiac disease (CeD) was 2.7% greater than the global pooled prevalence of 1.4%. Patients who strictly adhere to a lifetime gluten-free diet (GFD) may develop nutritional deficiencies potentially contributing to obesity, increased cardiovascular risk, and lower bone density. Therefore, this study aims to assess the knowledge of health students in the Jazan region regarding nutritional deficiencies in patients with CeD who are on a GFD and to determine the associated socio-demographic factors.

Methods: A descriptive cross-sectional study was conducted among health college students of Jazan University, including students from the College of Medicine, Pharmacy, Nursing, Dentistry, Public Health and Health Informatics, and Allied Health Sciences, aged 18 and above, excluding internship students, students who did not complete the survey, and those who refused to participate. The minimum calculated sample size was 368. The questionnaire was adopted from the literature and translated into Arabic. It contained a socio-demographic section and a knowledge section that included 12 questions focusing on the content of macro- and micronutrients in a GFD and the impact of the diet on the health of patients with CeD.

Results: The study included 369 participants, with 235 (64%) aged 17-22, 280 (76%) being females, and 341 (92%) being single. The College of Medicine and the College of Applied Medical Sciences had the highest representation, and the College of Dentistry was the lowest. Most participants were in the fourth year (30%) and sixth year (24%). The grade point average (GPA) had a median of 4.3. Approximately 59.1% were aware of nutritional deficiencies among CeD patients. None of the sociodemographic factors were associated with health students' knowledge regarding CeD. Participants from the College of Pharmacy had a lower knowledge of CeD nutrient deficiencies than those from the College of Medicine. (OR: 0.45, 95% CI: 0.22, 0.90)*. *Most students stated that CeD patients should be advised to take multivitamin drugs and vitamin D supplements. Most acknowledged vitamin D, vitamin B12, and folic acid deficiencies in CeD patients. Similarly, most were aware of iron and calcium deficiencies, with a small percentage aware of zinc and copper.

Conclusion: Approximately 59.1% had an acceptable level of knowledge, which is considered a low knowledge level among students who will be future physicians and healthcare workers to whom CeD will represent the first-line disease exposed to. Rising knowledge and awareness among those students will guarantee correct diagnosis, treatment, and better outcomes among CeD patients, thus decreasing the disease burden and increasing the quality of those patients.

## Introduction

Celiac disease (CeD) is a systemic, small intestinal, immune-mediated enteropathy triggered and maintained by gluten proteins present in cereals like wheat, barley, and rye [1]. Historically perceived as uncommon and confined to specific European regions [2], a global systematic review and meta-analysis, encompassing 275,818 subjects, revealed a pooled seroprevalence of CeD at 1.4%, with biopsy-confirmed prevalence at 0.7%, signifying one in 140 individuals affected [3]. In Asian countries, a recent meta-analysis reported a serological prevalence of 1.6% among 47,873 participants, with biopsy-proven CeD at 0.5% in 43,955 individuals [4]. Stratification by quartiles highlighted variations in prevalence across countries [5]. In Saudi Arabia, the prevalence of biopsy-proven CeD was 1.4% while the seroprevalence was 2.7% [6].

The long-term consequences of CeD involve malabsorption of essential nutrients such as iron, folic acid, vitamins B6 and B12, vitamin D, copper, and zinc [7]. The 2013 American College of Gastroenterology guidelines affirmed the frequent occurrence of micronutrient deficiencies in celiac patients at diagnosis, emphasizing the importance of early detection and integration [7]. Pediatric CeD patients are at risk of osteoporosis, anemia, stunted growth, short stature, and delayed puberty [8]. Increased risks of subsequent hip fracture and fractures of any kind were observed, independent of age or sex [9,10].

A lifetime gluten-free diet (GFD) is the primary treatment for CeD [11]. Strict adherence is clinically effective, but recent perceptions of gluten-free products as “very healthy” have led to widespread consumption by individuals without CeD [12]. Non-adherence to the GFD increases the risk of morbidity and mortality, including infertility, skeletal disorders, and malignancy [11]. However, studies suggest a potential association between GFD and nutritional deficiencies [13,14]. A systematic review revealed that children with CeD following a GFD may be at risk of excessive fat consumption and insufficient intake of fiber, iron, vitamin D, and calcium [13]. Alterations in folate, magnesium, and zinc intake were also noted, potentially contributing to obesity, increased cardiovascular risk, and lower bone density, particularly crucial in the developmental age [15,16]. Hence, this study aims to assess the knowledge of health students in the Jazan region regarding nutritional deficiencies in patients with CED on a GFD and to determine the associated socio-demographic factors.

## Materials and methods

Study design, setting, and population

A descriptive cross-sectional study was conducted between July 2023 and December 2023 among health college students of Jazan University aged 18 and above, excluding internship students, students who did not complete the survey, and those who refused to participate.

Sample size and data collection process

The sample size was calculated using the Raosoft calculator (Raosoft, Inc., Seattle, WA) [17]. Considering a 95% confidence interval, a 5% margin of error, a 50% population proportion, a 25% non-response rate, and a health student population of 8,424, the calculated sample was 368. The data were collected through a convenient sampling technique using a self-administered electronic questionnaire that was distributed in a Google form to different social media platforms among all students meeting the inclusion criteria. The questionnaire was adopted from the literature [18] and translated into Arabic. The questionnaire was reviewed and approved; a pilot study was conducted among 29 participants (10% of the sample size) to test the clarity, understandability, and accuracy of the questionnaire. Participant feedback from this preliminary phase was used to improve the final survey. The questionnaire took about three to five minutes to complete, and it was divided into two sections. The first section contained questions regarding socio-demographic data like age, gender, nationality, residency, marital status, monthly income, the college the participant studies, academic year, and Grade point average (GPA). The second section focused on the knowledge of nutritional deficiencies and preventing such deficiencies in CeD patients who are on a GFD. The survey contained 12 single or multiple-choice questions that focused on the content of macro- and micronutrients in a GFD and the impact of the diet on the health of patients with CD.

Statistical analysis

Statistical data analysis was performed using SPSS software version 27 (IBM Corp., Armonk, NY). Means and standard deviations were used to summarize continuous variables, while frequencies and percentages were used to represent categorical variables. The correct answers were coded one, and incorrect ones were coded zero and the total score was calculated. To categorize the knowledge, we considered those who answered 60% (⅝) of correct answers as having “acceptable knowledge.” The chi-square test was utilized to assess the determinants of CeD knowledge. Multiple logistic regression was used to determine the predictors of CeD knowledge. A p-value of less than 0.05 was set as the significance level. 

Ethical consideration

The ethical approval was obtained from the Scientific Research Ethics Committee (REC) at Jazan University, Saudi Arabia, under reference number (REC-44/11/704). Each participant's informed consent was obtained and their right to withdraw at any moment was assured. Privacy and confidentiality were preserved.

## Results

The study included 369 participants, with 64% (235) aged 17-22, 76% (280) being females, and 98% being Saudi. Most of the students were from the College of Medicine 234 (63%), College of Applied Medical Sciences 54 (15%), College of Dentistry 10 (2.7%), College of Nursing 16 (4.3%), College of Pharmacy 44 (12%), and College of Public Health and Health Informatics 11 (3.0%). Most participants were in the fourth year 109 (30%). The GPA had a median of 4.30 and interquartile range of 4-4.7 (Table 1).

**Table 1:** General characteristics of study participants ^1^n (%); Median (IQR) SAR: Saudi Riyals (1 Saudi Riyal is equivalent to 0.266 dollars), GPA: Grade point average

Characteristic	N = 369^1^
Age	
17-22	235 (64%)
23 and above	134 (36%)
Gender	
Female	280 (76%)
Male	89 (24%)
Nationality	
Non-Saudi	7 (1.9%)
Saudi	362 (98%)
Residence	
Urban	219 (59%)
Rural	150 (41%)
Marital status	
Married	28 (7.6%)
Single	341 (92%)
Monthly income	
less than 5,000 SAR	232 (63%)
5,000-9,999 SAR	43 (12%)
10,000-20,000 SAR	48 (13%)
More than 20,000 SAR	46 (12%)
College	
College of Medicine	234 (63%)
College of Applied Medical Sciences	54 (15%)
College of Dentistry	10 (2.7%)
College of Nursing	16 (4.3%)
College of Pharmacy	44 (12%)
College of Public Health and Health Informatics	11 (3.0%)
Academic year	
First year (preparatory year)	7 (1.9%)
Second year	18 (4.9%)
Third year	75 (20%)
Fourth year	109 (30%)
Fifth year	71 (19%)
Sixth year	89 (24%)
GPA	4.30 (4.00, 4.70)
Missing	12

When examining the dietary recommendations for patients with CeD, most students 310 (84%) expressed that CeD patients should adhere to a GFD with an increased calorie content compared to their healthy counterparts. In addressing concerns about potential nutritional deficiencies in patients with CeD following a GFD, 60% (221) of the students acknowledged this risk. In comparison, 47% (174) agreed that CeD patients who are on a GFD can become overweight or obese. Exploring perceptions about a GFD's impact on carbohydrate consumption, 82% (304) of students believed that such a diet favors eating fewer complex carbohydrates. Approximately 50% did not agree that gluten-free processed foods contain more saturated fat than their gluten-containing counterparts. However, 65% (239) of students believed that gluten-free processed foods contain more dietary fiber than their gluten-containing counterparts; similarly, 46% (169) of students believed that gluten-free processed foods had a higher glycemic index than their gluten-containing counterparts. Finally, 81% (298) of students were aware of the necessity for regular vitamin D level assessments for all CeD patients regardless of supplementation. Students from the College of Medicine had a higher percentage of correct answers compared to other students thus higher awareness of CeD (Table 2).

**Table 2:** Knowledge of celiac disease among health students ^1^n (%)

Characteristic	Overall, N = 369^1^	College of Medicine, N = 234^1^	College of Nursing, N = 16^1^	College of Pharmacy, N = 44^1^	Others, N = 75^1^
Patients with celiac disease should follow a gluten-free diet that contains more calories compared to healthy people.					
No	59 (16%)	40 (68%)	3 (5.1%)	6 (10%)	10 (17%)
Yes*	310 (84%)	194 (63%)	13 (4.2%)	38 (12%)	65 (21%)
Patients with celiac disease can be exposed to nutritional deficiencies by following a gluten-free diet.					
No	148 (40%)	91 (61%)	5 (3.4%)	19 (13%)	33 (22%)
Yes*	221 (60%)	143 (65%)	11 (5.0%)	25 (11%)	42 (19%)
Individuals with celiac disease who follow a gluten-free diet can become overweight or obese					
No	195 (53%)	124 (64%)	6 (3.1%)	26 (13%)	39 (20%)
Yes*	174 (47%)	110 (63%)	10 (5.7%)	18 (10%)	36 (21%)
A gluten-free diet favors eating fewer complex carbohydrates					
No	65 (18%)	37 (57%)	7 (11%)	8 (12%)	13 (20%)
Yes*	304 (82%)	197 (65%)	9 (3.0%)	36 (12%)	62 (20%)
Gluten-free processed foods contain more saturated fat than their gluten-containing counterparts					
No*	184 (50%)	131 (71%)	7 (3.8%)	21 (11%)	25 (14%)
Yes	185 (50%)	103 (56%)	9 (4.9%)	23 (12%)	50 (27%)
Gluten-free processed foods contain more dietary fiber than their gluten-containing counterparts.					
No	130 (35%)	88 (68%)	8 (6.2%)	11 (8.5%)	23 (18%)
Yes*	239 (65%)	146 (61%)	8 (3.3%)	33 (14%)	52 (22%)
The glycemic index of gluten-free processed foods is higher compared with their gluten-containing counterparts					
No	200 (54%)	126 (63%)	5 (2.5%)	27 (14%)	42 (21%)
Yes*	169 (46%)	108 (64%)	11 (6.5%)	17 (10%)	33 (20%)
All patients with celiac disease should have regular assessment of vitamin D levels, regardless of their supplementation					
No	71 (19%)	38 (54%)	3 (4.2%)	9 (13%)	21 (30%)
Yes*	298 (81%)	196 (66%)	13 (4.4%)	35 (12%)	54 (18%)

None of the sociodemographic factors were associated with health students' knowledge regarding CeD (Table 3).

**Table 3:** Determinants of celiac disease knowledge ^1^n (%); Median (IQR) SAR: Saudi Riyal, GPA: Grade point average ^2^Pearson's Chi-squared test; Fisher's exact test; Wilcoxon rank sum test

Characteristic	Unacceptable knowledge, N = 151^1^	Acceptable knowledge, N = 218^1^	p-value^2^
Age			0.9
17-22	97 (64%)	138 (63%)	
23 and above	54 (36%)	80 (37%)	
Gender			0.5
Female	117 (77%)	163 (75%)	
Male	34 (23%)	55 (25%)	
Nationality			>0.9
Non-Saudi	3 (2.0%)	4 (1.8%)	
Saudi	148 (98%)	214 (98%)	
Residence			0.5
Urban	93 (62%)	126 (58%)	
Rural	58 (38%)	92 (42%)	
Marital status			0.9
Married	11 (7.3%)	17 (7.8%)	
Single	140 (93%)	201 (92%)	
Monthly income			0.8
less than 5000 SAR	96 (64%)	136 (62%)	
5000-9999 SAR	20 (13%)	23 (11%)	
10000-20000 SAR	18 (12%)	30 (14%)	
more than 20000 SAR	17 (11%)	29 (13%)	
College			0.3
College of Medicine	90 (60%)	144 (66%)	
College of Applied Medical Sciences	23 (15%)	31 (14%)	
College of Dentistry	2 (1.3%)	8 (3.7%)	
College of Nursing	7 (4.6%)	9 (4.1%)	
College of Pharmacy	24 (16%)	20 (9.2%)	
College of Public Health and Health Informatics	5 (3.3%)	6 (2.8%)	
Academic year			0.7
First year (preparatory year)	3 (2.0%)	4 (1.8%)	
Second year	7 (4.6%)	11 (5.0%)	
Third year	35 (23%)	40 (18%)	
Fourth-year	38 (25%)	71 (33%)	
Fifth year	29 (19%)	42 (19%)	
Sixth year	39 (26%)	50 (23%)	
GPA	4.30 (4.00, 4.72)	4.28 (3.99, 4.66)	0.7
Missing	7	5	

Participants from the College of Pharmacy had a lower knowledge of CeD nutrient deficiencies than those from the College of Medicine (OR: 0.45, 95% CI: 0.22, 0.90) (Table 4). 

**Table 4:** Determinants of celiac disease knowledge ^1^OR = Odds Ratio, CI = Confidence Interval

Characteristic	OR^1^	95% CI^1^	p-value
Gender			
Female	—	—	
Male	1.00	0.60, 1.69	>0.9
Residence			
Urban	—	—	
Rural	1.30	0.83, 2.04	0.3
College			
College of Medicine	—	—	
College of Applied Medical Sciences	0.80	0.43, 1.51	0.5
College of Dentistry	1.97	0.43, 13.9	0.4
College of Nursing	0.75	0.27, 2.17	0.6
College of Pharmacy	0.45	0.22, 0.90	0.024
College of Public Health and Health Informatics	0.55	0.15, 2.10	0.4
GPA	1.07	0.73, 1.55	0.7

**Figure 1:**
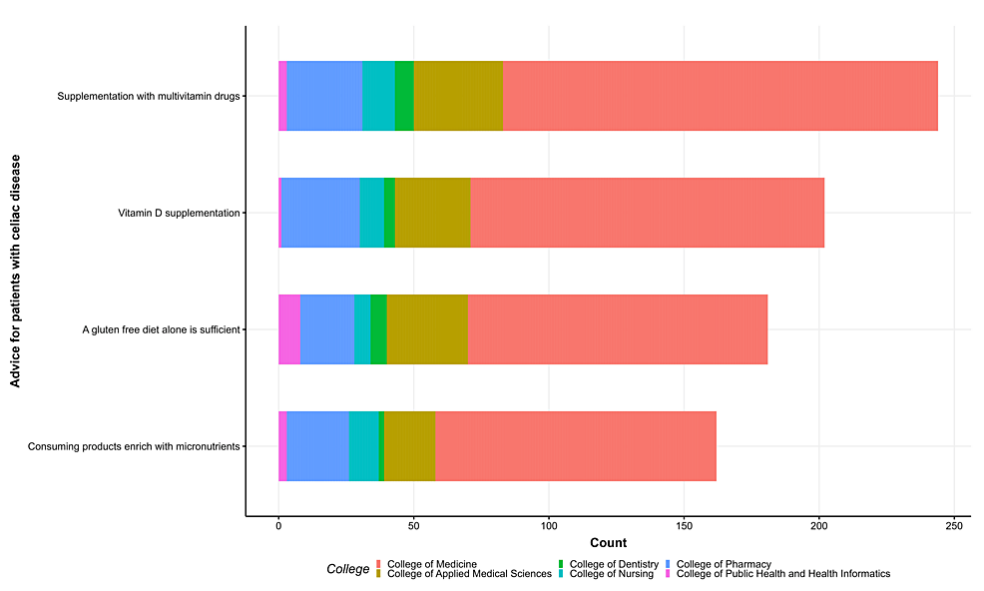
Knowledge of celiac disease vitamin deficiencies among health students in the Jazan

**Figure 2:**
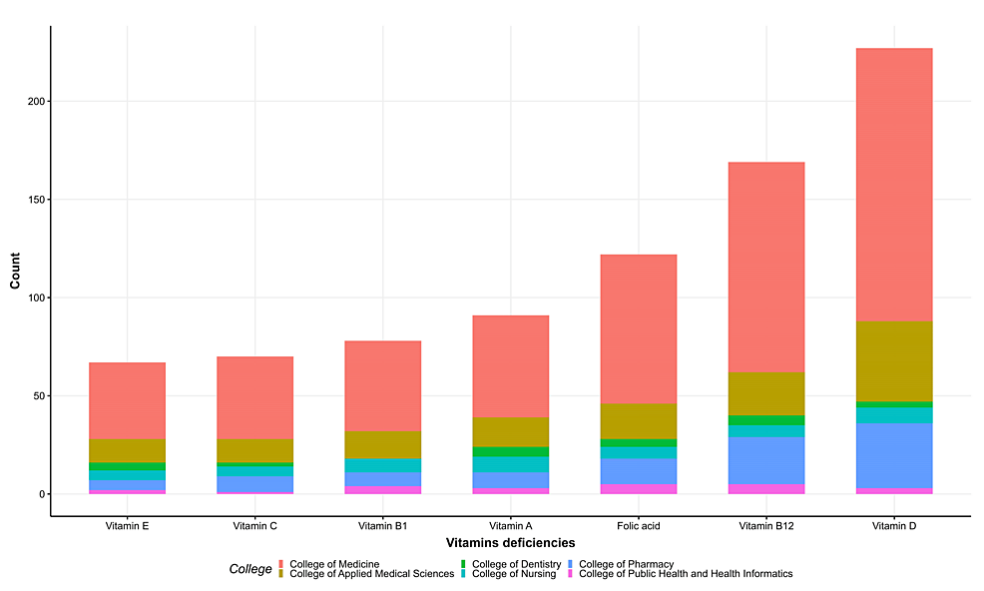
Knowledge of celiac disease vitamin deficiencies among health students in the Jazan

**Figure 3:**
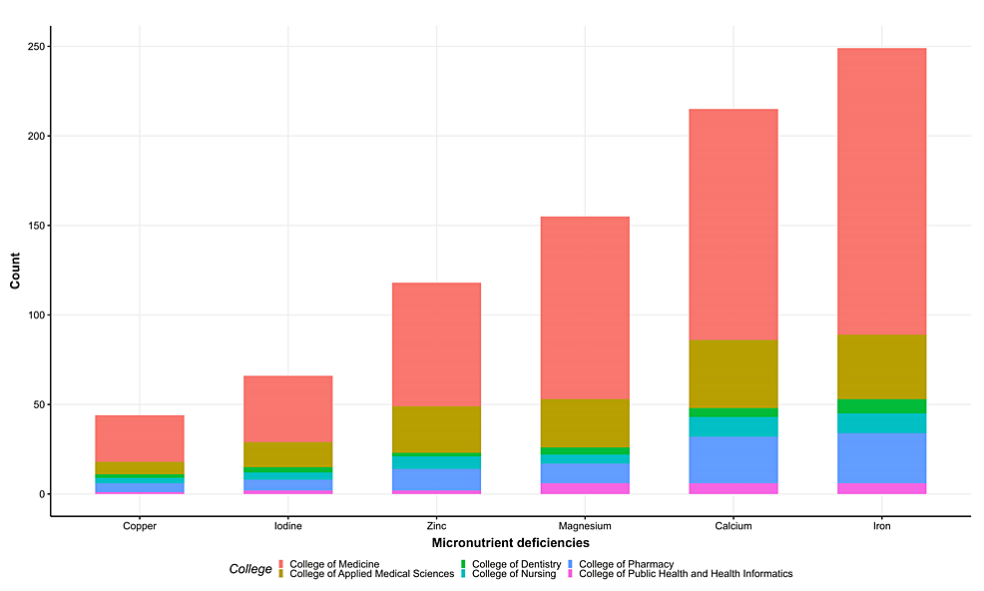
Knowledge of celiac disease micronutrient deficiencies among health students in the Jazan

**Figure 4:**
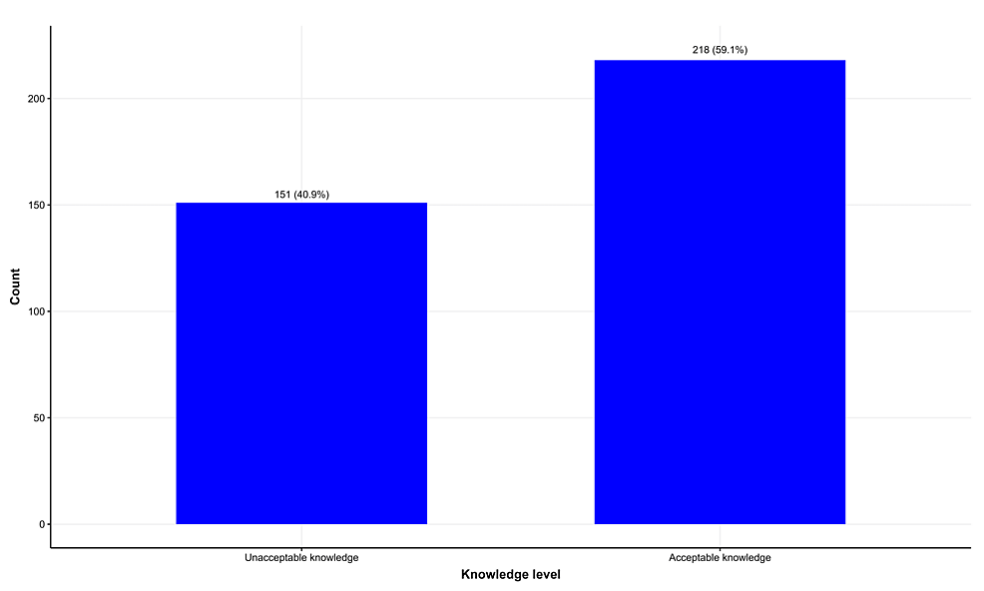
Knowledge of celiac disease among health students in the Jazan

Most students stated that CeD patients should be advised to take multivitamin drugs and vitamin D supplements (Figure 1), and most of them acknowledged vitamin D, vitamin B12, and folic acid deficiencies in CeD patients (Figure 2). Similarly, most were aware of iron and calcium deficiencies, with a small percentage aware of zinc and copper (Figure 3). Approximately 59.1% were aware of nutritional deficiencies among CeD patients (Figure 4).

## Discussion

This study, which included 369 participants, aimed to assess knowledge about CeD among medical sciences students in Saudi Arabia. Most participants were in the age group 17-22 years; 76% (280) were females 98% were of Saudi nationality, and 63% (232) had less than 5,000 SAR monthly income. Regarding college enrollment, 63% (234) were from the College of Medicine, 15% (54) were from the College of Applied Medical Sciences, 12% (44) from the College of Pharmacy, and the remaining were from dentistry, nursing, and public health and health informatics colleges. Upon assessment of the overall knowledge level among our participants, 59.1% (218) had an acceptable level of knowledge about CeD. Assiri et al. assessed CeD knowledge among Saudi physicians; 56.8% had good knowledge, while 19.2% had poor knowledge. Interestingly, younger physicians were more knowledgeable; for instance, interns had more knowledge than consultants [19]. In Kazakhstan, 59.4% had poor knowledge of CeD. Nevertheless, physicians with an experience period of less than three years were more knowledgeable [20]. In Iran, 63.5% of physicians were found to have intermediate knowledge, 24.4% have good knowledge, and 12.2% have a weak level of knowledge [21]. A study among dentistry students in India revealed that 44.5% of students were aware of CeD [22]. In Europe, the knowledge level of CeD among healthcare professionals was found to be a mean of 50.9%. In comparison, the mean knowledge score among pediatric gastroenterologists was 69.4%, indicating discrepancy within the medical field between professionals, as pediatric gastroenterologists are the most likely to encounter patients with CeD [23,24]. However, in Romania, Mariana et al. revealed that there is a poor level of knowledge among physicians regarding clinical features and diagnostic measurements for CeD [25]. Among medical students, 46% of them were not aware of nutritional deficiencies in CeD; among healthcare professionals, dietitians were the most knowledgeable about nutritional disorders in CeD patients [18]. As dietitians are a critical part of CeD assessment and treatment, many studies have assessed their awareness of CeD and its nutritional aspects. In Australia, dietitians had a 73% score in the knowledge about appropriate food for CeD and 69% in the knowledge related to screening and diagnosis of the disease [26]. In the United States, 85% of dietitian nutritionists were knowledgeable when assessed in five knowledge questions [27].

It is quite vital that students in the medical field should be well educated about CeD, as inadequate awareness of future physicians about this disease can lead to misdiagnosis and under-treatment of CeD [28]. When assessing determinants of CeD knowledge among health students, none of the sociodemographic factors was statistically significant. However, this study possesses limitations. This was a cross-sectional study limited by convenience sampling. The study's inability to adequately justify the reason for the observed differences in knowledge levels among college students. Lastly, as this study used an online survey to collect data, the impact of recall and selection bias cannot be disregarded.

## Conclusions

To conclude this study, which assessed the knowledge about CeD among medical field students, 59.1% (218) had an acceptable level of knowledge. This could be considered a low knowledge level among students from fields that future physicians and healthcare workers who represent the first-line CeD will be exposed to. Rising knowledge and awareness among those students will guarantee correct diagnosis, treatment, and better outcomes among CeD patients, thus decreasing the disease burden and increasing the quality of this chronic disease. More educational programs are recommended to be conducted for medical, pharmacy, and dentistry students and students from other related medical fields. Medical education authorities should make more efforts to empower students in the medical field with all necessary knowledge to decrease rates of disease miss-diagnosis and under-treatment. These programs should not only teach about CeD but also incorporate practical aspects such as diagnostic approaches, modalities, and management strategies. Moreover, curriculum involvement and teaching topics should be carefully considered and integrated into these educational initiatives.
